# Outcome of liver cancer patients with SARS‐CoV‐2 infection: An International, Multicentre, Cohort Study

**DOI:** 10.1111/liv.15320

**Published:** 2022-06-23

**Authors:** Sergio Muñoz‐Martínez, Victor Sapena, Alejandro Forner, Jordi Bruix, Marco Sanduzzi‐Zamparelli, José Ríos, Mohamed Bouattour, Mohamed El‐Kassas, Cassia R. G. Leal, Tudor Mocan, Jean‐Charles Nault, Rogerio C. P. Alves, Helen L. Reeves, Leonardo da Fonseca, Ignacio García‐Juárez, David J. Pinato, María Varela, Saleh A. Alqahtani, Mario R. Alvares‐da‐Silva, Juan C. Bandi, Lorenza Rimassa, Mar Lozano, Jesús M. González Santiago, Frank Tacke, Margarita Sala, María Anders, Anja Lachenmayer, Federico Piñero, Alex França, Maria Guarino, Alessandra Elvevi, Giuseppe Cabibbo, Markus Peck‐Radosavljevic, Ángela Rojas, Mercedes Vergara, Chiara Braconi, Sonia Pascual, Christie Perelló, Vivianne Mello, Carlos Rodríguez‐Lope, Juan Acevedo, Rosanna Villani, Clemence Hollande, Valérie Vilgrain, Ahmed Tawheed, Carmem Ferguson Theodoro, Zeno Sparchez, Lorraine Blaise, Daniele E. Viera‐Alves, Robyn Watson, Flair J. Carrilho, Carlos Moctezuma‐Velázquez, Antonio D'Alessio, Massimo Iavarone, Maria Reig

**Affiliations:** ^1^ Unitat d'Oncologia hepàtica, Liver Unit, Hospital Clínic Barcelona Spain; ^2^ BCLC group, IDIBAPS Barcelona Spain; ^3^ CIBEREHD Barcelona Spain; ^4^ Universitat de Barcelona (UB) Barcelona Spain; ^5^ Medical Statistics Core Facility, Institut D'Investigacions Biomédiques August Pi i Sunyer (IDIBAPS), Hospital Clinic Barcelona Barcelona Spain; ^6^ Department of Clinical Farmacology Hospital Clinic and Medical Statistics Core Facility, Institut d'Investigacions Biomèdiques August Pi i Sunyer (IDIBAPS) Biostatistics Unit, Faculty of Medicine, Universitat Autònoma de Barcelona Barcelona Spain; ^7^ AP‐HP, Hôpital Beaujon, Liver Cancer Unit Clichy France; ^8^ Endemic Medicine Department Faculty of Medicine Helwan University Cairo Egypt; ^9^ Gastroenterology Hospital Universitário Antônio Pedro, Universidade Federal Fuminense Rio de Janeiro Brazil; ^10^ Gastroenterology Hospital Federal do Servidores do Estado Rio de Janeiro Brazil; ^11^ 3rd Medical Department "Octavian Fodor" Institute for Gastroenterology and Hepatology Cluj‐Napoca Romania; ^12^ Liver unit Hôpital Avicenne, Hôpitaux Universitaires Paris‐Seine‐Saint‐Denis, Assistance‐Publique Hôpitaux de Paris Bobigny France; ^13^ Unité de Formation et de Recherche Santé Médecine et Biologie Humaine Université Paris Nord, Communauté d'Universités et Etablissements Sorbonne Paris Cité Paris France; ^14^ Centre de Recherche des Cordeliers Sorbonne Université Inserm, Equipe labellisée Ligue Nationale Contre le Cancer, Labex OncoImmunology. Université de Paris, team « Functional Genomics of Solid Tumors Paris France; ^15^ Gastroenterology Hospital do Servidor Publico Estadual de São Paulo Sao Paulo Brazil; ^16^ Bp Beneficência Portuguesa de São Paulo Sao Paulo Brazil; ^17^ Liver Unit Newcastle Hospitals NHS Foundation Trust Newcastle upon Tyne UK; ^18^ Newcastle University Translational and Clinical Research Institute Newcastle upon Tyne UK; ^19^ Clinical Oncology Sao Paulo Clinicas Liver Cancer Group. Instituto do Cancer do Estado de Sao Paulo. Hospital das Clínicas. University of Sao Paulo School of Medicine Sao Paulo Brazil; ^20^ Gastroenterology Department National Institute of Medical Sciences and Nutrition Salvador Zubirán Mexico City Mexico; ^21^ Department of Surgery and Cancer Imperial College London London UK; ^22^ Liver Unit, Department of Digestive Disease Hospital Universitario Central de Asturias, IUOPA, ISPA, Universidad de Oviedo Oviedo Spain; ^23^ Liver Transplant King Faisal Specialist Hospital & Research Center Riyadh Saudi Arabia; ^24^ GI/Liver Unit Hospital de Clínicas de Porto Alegre, Universidade Federal do Rio Grande do Sul Porto Alegre Brazil; ^25^ Hepatology Unit Hospital Italiano Buenos Aires Argentina; ^26^ Department of Biomedical Sciences Humanitas University Rozzano Italy; ^27^ Medical Oncology and Hematology Unit Humanitas Cancer Center, IRCCS, Humanitas Research Hospital Rozzano Italy; ^28^ Aparato Digestivo Hospital Universitario Infanta Leonor Madrid Spain; ^29^ Department of Gastroenterology and Hepatology Salamanca University Clinic Hospital IBSAL, CIBERehd Salamanca Spain; ^30^ Department of Hepatology and Gastroenterology Charité‐Universitätsmedizin Campus Virchow‐Klinikum and Campus Charité Mitte Berlin Germany; ^31^ Gastroenterology Hepatology Unit Hospital Doctor Josep Trueta, IDIBGI (Institut d'Investigació Biomèdica de Girona) CIBERehd Girona Spain; ^32^ Hospital Aleman Hepatología Buenos Aires Argentina; ^33^ Department of Visceral Surgery and Medicine Inselspital, Bern University Hospital, University of Bern Bern Switzerland; ^34^ Liver Unit Hospital Universitario Austral Pilar Argentina; ^35^ Medicine Federal University of Sergipe Aracaju Brazil; ^36^ Department of Clinical Medicine and Surgery University of Naples Federico II Napoli Italy; ^37^ Division Gastroenterology and Center for Autoimmune Liver Diseases San Gerardo Hospital University of Milano ‐ Bicocca School of Medicine Monza Italy; ^38^ Section of Gastroenterology and Hepatology PROMISE, University of Palermo Palermo Italy; ^39^ Innere Medizin & Gastroenterologie, Klinikum Klagenfurt am Wörthersee Klagenfurt Austria; ^40^ SeLiver group. Institute of Biomedicine of Seville Hospital Universitario Virgen del Rocío/CSIC/Universidad de Sevilla‐CIBERehd Seville Spain; ^41^ Unitat d'Hepatologia, Servei d'Aparell Digestiu, Parc Taulí Sabadell Hospital Universitari, Institut d'Investigació i Innovació I3PT, Universitat Autònoma de Barcelona, Barcelona, Departament de Medicina, Universitat Autònoma de Barcelona, CIBERehd. Instituto Carlos III Madrid Spain; ^42^ Medical Oncology Beatson West of Scotland Cancer Centre University of Glasgow Glasgow UK; ^43^ Liver Unit. HGU Dr. Balmis. CIBERehd. ISABIAL Alicante Spain; ^44^ Gastroenterology and Hepatology University Hospital Puerta de Hierro Majadahonda Spain; ^45^ Oncology AMO CLINIC Salvador Brazil; ^46^ Servicio de Aparato Digestivo Hospital Universitario Marqués de Valdecilla IDIVAL Santander Spain; ^47^ South West Liver Unit University Hospitals Plymouth NHS Trust Plymouth UK; ^48^ Department of Medical and Surgical Sciences Foggia Italy; ^49^ AP‐HP Hôpital Beaujon, Liver Cancer Unit Clichy France; ^50^ Université de Paris Paris France; ^51^ Department of Radiology Hôpital Beaujon, AP‐HP. Nord Clichy France; ^52^ Department of Gastroenterology Hospital Universitário Antônio Pedro, Universidade Federal Fluminense Niteroi Brazil; ^53^ 3rd Medical Department Institute for Gastroenterology and Hepatology University of Medicine and Pharmacy Cluj‐Napoca Romania; ^54^ Sao Paulo Clínicas Liver Cancer Group. Instituto do Cancer do Estado de Sao Paulo. Division of Clinical Gastroenterology and Hepatology Hospital das Clínicas, Department of Gastroenterology University of Sao Paulo School of Medicine Sao Paulo Brazil; ^55^ Foundation IRCCS Ca' Granda Ospedale Maggiore Policlinico – Division of Gastroenterology and Hepatology Milan Italy

**Keywords:** COVID‐19, hepatocellular carcinoma, liver cancer, mortality

## Abstract

**Background & Aims:**

Information about the impact of severe acute respiratory syndrome coronavirus 2 (SARS‐CoV‐2) in patients with liver cancer is lacking. This study characterizes the outcomes and mortality risk in this population.

**Methods:**

Multicentre retrospective, cross‐sectional, international study of liver cancer patients with SARS‐CoV‐2 infection registered between February and December 2020. Clinical data at SARS‐CoV‐2 diagnosis and outcomes were registered.

**Results:**

Two hundred fifty patients from 38 centres were included, 218 with hepatocellular carcinoma (HCC) and 32 with intrahepatic cholangiocarcinoma (iCCA). The median age was 66.5 and 64.5 years, and 84.9% and 21.9% had cirrhosis in the HCC and iCCA cohorts respectively. Patients had advanced cancer stage at SARS‐CoV‐2 diagnosis in 39.0% of the HCC and 71.9% of the iCCA patients. After a median follow‐up of 7.20 (IQR: 1.84–11.24) months, 100 (40%) patients have died, 48% of the deaths were SARS‐CoV‐2‐related. Forty (18.4%) HCC patients died within 30‐days. The death rate increase was significantly different according to the BCLC stage (6.10% [95% CI 2.24–12.74], 11.76% [95% CI 4.73–22.30], 20.69% [95% CI 11.35–31.96] and 34.52% [95% CI 17.03–52.78] for BCLC 0/A, B, C and D, respectively; *p* = .0017). The hazard ratio was 1.45 (95% CI 0.49–4.31; *p* = .5032) in BCLC‐B versus 0/A, and 3.13 (95% CI 1.29–7.62; *p* = .0118) in BCLC‐C versus 0/A in the competing risk Cox regression model. Nineteen out of 32 iCCA (59.4%) died, and 12 deaths were related to SARS‐CoV‐2 infection.

**Conclusions:**

This is the largest cohort of liver cancer patients infected with SARS‐CoV‐2. It characterizes the 30‐day mortality risk of SARS‐CoV‐2 infected patients with HCC during this period.

**Abbreviations**
BCLC: Barcelona clinic liver cancer
BSC: best supportive care
CI: confidence interval
COVID‐19: coronavirus disease 2019
HCC: hepatocellular carcinoma
HR: Hazard ratio
iCCA: intrahepatic cholangiocarcinoma
IQR: interquartile range
SARS‐CoV‐2: severe acute respiratory syndrome

**Keypoints**

Data regarding clinical profile and outcomes of liver cancer patients with SARS‐CoV‐2 infection were lacking. This international project aims to characterize these patients´ outcomes and generate clinical data useful for informed prognosis prediction in this population.

## 1 INTRODUCTION

After the start of the Coronavirus Disease 2019 (COVID‐19) in 2019, all countries worldwide made a huge effort to face up to the health issues derived from the pandemic. In December 2020 the first SARS‐CoV‐2 vaccine was authorized by the U.S. Food and Drug Administration, while it was granted a conditional marketing authorization by the European Medicines Agency.^1^ Nevertheless, just after the first wave, further waves emerged and the sequelae of the pandemic will probably continue for years. Our previous study^2^ showed that all treatments, except systemic therapy, had relevant interruptions during the first wave around the World. Indeed, 48% of the centres decreased the number of physicians devoted to managing liver cancer. Gandhi et al.^3^ also assessed the impact on COVID‐19 in 14 Asia‐Pacific countries and observed similar results. One of the main harms of the pandemic according to Muñoz et al.^2^ was the delay in liver cancer diagnosis because of the modification of screening, reported in 80.9% of the participating centres. A similar impact of COVID‐19 was also reported for other cancers^4^, ^5^, ^6^, ^7^ and in the current study, here we characterize the profile and evolution of those patients incidentally diagnosed with liver cancer as a result of the assessments done because of COVID‐19 infection diagnosis and those who had a history of liver cancer.

A microsimulation model on five cancers (breast, cervix, colorectal, prostate and stomach) found that delays in diagnosis will result in a worse cancer stage at presentation, leading to worse survival outcomes.^8^ Liver cancer was not represented in that model and such data should be confirmed in the liver cancer realm. A second harm of the pandemic is the COVID‐19‐related and non‐COVID‐19‐related mortality.^9^ In the liver cancer setting, the mortality analysis is complex because almost all hepatocellular carcinoma (HCC) patients and some of the intrahepatic cholangiocarcinoma (iCCA) patients present underlying cirrhosis.

Iavarone et al.^10^ evaluated the 30‐day mortality rate in cirrhotic patients but only 22% of them had active or history of liver cancer. Thus, there is neither mortality data nor information about the impact of the liver cancer stage in the outcome of patients diagnosed as a result of SARS‐CoV‐2 diagnosis. Lai et al.^11^ analysed the indirect excess deaths (because of pandemic‐induced healthcare service reconfiguration) on cancer patients from the United Kingdom. They concluded that cancer services had only partially recovered with the lockdown easing. They also suggested that this situation may contribute to substantial excess mortality and multimorbidity among cancer patients. According to their analysis, the 1‐year liver cancer mortality in patients without comorbidities or with one or two comorbidities are 50.2%, 50.3% and 49.5% respectively. Here, again there is neither information about the liver cancer stage nor the impact of the 30‐day mortality rate. They pointed out the urgent need to better understand and mitigate these excess mortality risks. The present analysis is the second part of the Liver cancer outcome in the COVID‐19‐pandemic (CERO‐19) which aims to address the outcome of SARS‐CoV‐2 on liver cancer patients and to understand the confounding factors at the time of analysing their mortality.

The specific aims of the present analysis were (1) to describe the profile of patients with liver cancer as a result of the tests performed because of SARS‐CoV‐2 infection as well as their outcome; (2) to analyse the 30‐day mortality rate of liver cancer patients with SARS‐CoV‐2 infection. This information will be key to understand the outcome of liver cancer patients who started oncologic treatments before or during the pandemic as well as the evolution of new liver cancer diagnosed during SARS‐CoV‐2 infection.

## 2 PATIENTS AND METHODS

### 2.1 Patients

This is a multicentre, retrospective, cross‐sectional and international study that evaluated the clinical outcomes of liver cancer patients diagnosed with SARS‐CoV‐2. Centres around the world were invited to participate as described in CERO‐19 project.^2^

The inclusion criteria were (1) patients older than 18 years old; (2) with de novo or history of HCC or iCCA and (3) who were infected with SARS‐CoV‐2 between February and December 2020.

SARS‐CoV‐2 diagnosis was defined according to each centre local policy: Positive result on a reverse‐transcription PCR (RT‐PCR) assay of a specimen collected on a nasopharyngeal swab, positive antigen test and/or radiological changes compatible with SARS‐CoV‐2 diagnosis in a patient with clinical signs of SARS‐CoV‐2 infection.

### 2.2 Data collection

The study was approved by the institutional review board (HCB/2020/0454). Each centre was responsible to obtain the local approval for the project in their centre. The study complied with the provision of the Good Clinical Practice guidelines and the Declaration of Helsinki.

The data registry started from the date of the first SARS‐CoV‐2 infection described in each country, allowing patient's inclusion from February 2020 until December 2020.

### 2.3 Variables

The study used REDCap® for data collection. Included patients were de‐identified and assigned to an individual‐anonymized alphanumeric code.

The clinical variables registered were the presence of cirrhosis (yes/no), Child‐Pugh status previous to and at SARS‐CoV‐2 infection, liver disease aetiology, date of SARS‐CoV‐2 diagnosis, liver cancer stage at the moment of SARS‐CoV‐2 diagnosis by BCLC staging^12^, ^13^ system for HCC patients and TNM‐8th edition staging system^14^ for iCCA, the last liver cancer treatment (if any) received before SARS‐CoV‐2 infection diagnosis, patient's liver cancer treatment after the resolution of the SARS‐CoV‐2 infection, if there was need to stop or delay the liver cancer treatment because of SARS‐CoV‐2 infection, and if there was liver cancer progression, specifying the date and pattern of the progression.

The centres specified for each patient if hospitalization because of SARS‐CoV‐2 diagnosis was needed, SARS‐CoV‐2 infection treatment (including use of antibiotics, anti‐thrombotic prophylaxis and corticosteroids), dates of start and end of the treatment and their outcome. The last follow‐up date until 30th June 2021 or death date were registered, specifying if death was SARS‐CoV‐2 infection‐related or not related, claryfing the cause in the latter.

### 2.4 Statistical analysis

Continuous or ordinal variables were expressed as median and interquartile range [IQR: 25th–75th percentiles]. Categorical data were expressed as absolute frequency and percentages (%).

The 30‐day mortality rate and their 95% confidence intervals (95% CI) were calculated using Kaplan–Meier method. The 30‐day SARS‐CoV‐2‐related death rate (or non‐SARS‐CoV‐2‐related death) was calculated with Kaplan–Meier method using the non‐SARS‐CoV‐2‐related death (or SARS‐CoV‐2‐related death) as competing risk. Cox regression models with non‐SARS‐CoV‐2‐related death as competing risks were used to estimate sub‐distribution Hazard Ratios (HR) and their 95% CI.

The level of significance was set at 5% (two‐sided). All statistical analyses were performed using SAS 9.4 software (SAS Institute).

## 3 RESULTS

### 3.1 Baseline characteristics

A total of 252 patients were registered. Two patients were excluded (one had a focal nodular hyperplasia and the second a non‐specified liver cancer different to HCC or iCCA). Therefore, 250 patients from 38 centres were included between February 1st, and December 31st, 2020. Table S1 describes the centres included in Europe, America, Asia and Africa.

**FIGURE 1:**
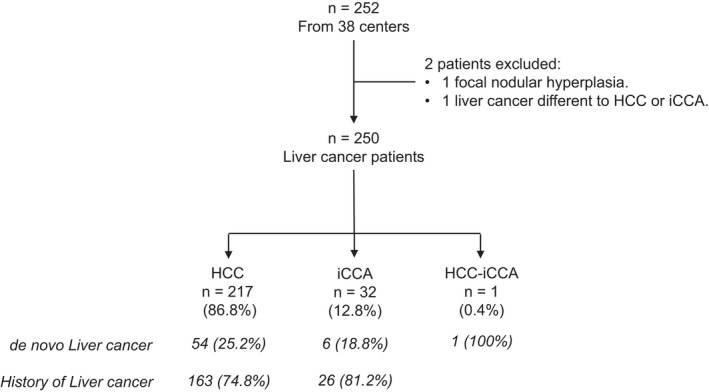
Flowchart of the study. During the inclusion period, 252 patients were registered; two patients were excluded and 250 were included in the study analysis. HCC, hepatocellular carcinoma; iCCA, intrahepatic cholangiocarcinoma.

Figure 1 describes the flow chart of the study. Sixty‐one (24.4%) patients had *de‐novo* liver cancer diagnosis (54 [90.2%] HCC and 6 [9.8%] iCCA), 163 (65.2%) had a history of HCC, and 26 (10.4%) had a history of iCCA. Only one patient was diagnosed with hepato‐cholangiocarcinoma (HCC‐iCCA).

The demographic and clinical characteristics of the patients are reported in Table 1. The median age was 66.5 [IQR 60–73] and 64.5 [IQR 57–74] years, 156 (71.6%) and 18 (56.3%) patients were male, 185 (84.9%) and 7 (21.9%) patients had cirrhosis in the HCC and iCCA cohorts respectively. The main aetiology was HCV (37.6%) in HCC patients and 62.5% of the iCCA patients had no liver disease history. One hundred and thirty‐nine (55.6%) patients were hospitalized because of SARS‐CoV‐2 and 108 (77.7%) of them received specific SARS‐CoV‐2 treatment according to the local medical practice.

**TABLE 1:** Baseline characteristics by liver cancer and outcome

Patient profile	HCC (*n* = 218)^a^	iCCA (*n* = 32)
Age (years), median [IQR]	66.5 [60–73]	64.5 [57–74]
Gender (Males), *n* (%)	156 (71.6)	18 (56.3)
Cirrhosis (Yes), *n* (%)	185 (84.9)	7 (21.9)
Child‐Pugh classification at SARS‐CoV‐2 diagnosis, *n* (%)		
A	104 (56.2)	3 (42.8)
B	63 (34.1)	2 (28.6)
C	17 (9.2)	2 (28.6)
Not available	1 (0.5)	‐
Non‐cirrhotic	33 (15.1)	25 (78.1)
Aetiology, *n* (%)		
HCV	82 (37.6)	4 (12.5)
Alcohol	44 (20.2)	3 (9.4)
NAFLD	38 (17.4)	3 (9.4)
HBV	19 (8.7)	‐
Alcohol and HCV	9 (4.1)	‐
Alcohol and NAFLD	7 (3.2)	‐
Combination of previous^b^	5 (2.3)	‐
Other	6 (2.8)^c^	2 (6.2)^d^
Non‐liver disease	6 (2.8)	20 (62.5)
Co‐infection HCV + HBV	2 (0.9)	‐
Liver cancer stage, *n* (%)	BCLC stage	TNM^e^
	0: 12 (5.5)	IA: 5 (15.6)
	A: 70 (32.1)	IB: 2 (6.3)
	B: 51 (23.4)	II: 2 (6.3)
	C: 58 (26.6)	IIIA: 1 (3.1)
	D: 27 (12.4)	IIIB: 8 (25)
		IV: 14 (43.7)
Liver cancer treatment received before SARS‐CoV‐2 diagnosis (liver cancer history patients), *n* (%)	163 (74.8)	26 (81.3)
Locoregional	77 (47.2)	‐
History of systemic treatment	44 (27)	19 (73.1)
Resection	20 (12.3)	3 (11.5)
Liver transplant	4 (2.5)	‐
BSC	17 (10.4)	2 (7.7)
None	1 (0.6)	1 (3.8)
Not specified	‐	1 (3.8)
Enrolled in a clinical trial (Yes), *n* (%)^f^	8 (16.3)	‐
Hospitalization due SARS‐CoV‐2 infection (Yes), *n* (%)	123 (56.4)	16 (50)
Received SARS‐CoV‐2 treatment (Yes), *n* (%)	101 (46.3)	7 (21.9)
Follow‐up time (days), median [IQR]	224 [70–352]	103 [12–266]
Deaths, *n* (%)	81 (37.2)	19 (59.4)
SARS‐CoV‐2 related deaths, *n* (%)	36 (44.4)	12 (63.2)
30‐day posterior to SARS‐CoV‐2 infection deaths, *n* (%)	40 (18.4)	12 (37.5)

Abbreviations: BCLC, Barcelona clinic liver cancer; HBV, hepatitis B virus; HCC, hepatocellular carcinoma; HCV, hepatitis C virus; iCCA, intrahepatic cholangiocarcinoma; IQR, interquartile range; NAFLD, non‐alcoholic fatty liver disease; SARS‐CoV‐2, severe acute respiratory syndrome coronavirus 2.

^a^ One patient with HCC‐iCCA.

^b^ Combination: NAFLD and HCV (1); NAFLD and HBV (1); Alcohol and HCV‐HBV co‐infection (1); HCV, NAFLD and autoimmune hepatitis (1); Graft‐versus‐host disease and Non‐alcoholic steatohepatitis (1).

^c^ Hemochromatosis (2), autoimmune hepatitis (2), biliary cholangitis (1), schistosomiasis (1).

^d^ NAFLD and biliary cirrhosis (1), Primary sclerosing cholangitis (1).

^e^ TNM 8th edition staging system of iCCA.

^f^ Percentage calculated from 49 patients that received systemic treatment.

One hundred (40%) patients died after a median follow‐up of 7.20 (IQR: 1.84–11.24) months, 48 (48%) were SARS‐CoV‐2‐related and 34 (70.1%) of them had cirrhosis. The other 52 (52%) patients died because of non‐SARS‐CoV‐2‐related causes and 86.5% of them were cirrhotic. One hundred and eight patients received treatment because of SARS‐CoV‐2 treatment, the most frequent were azithromycin (49.1%) and corticosteroids (42.6%), rest of the reported treatments are available in Table S2.

Fifty‐two patients (20.8%) died within the first 30 days of SARS‐CoV‐2 infection, and 43 (82.7%) of the deaths were SARS‐CoV‐2‐related. The 30‐day mortality rate in the whole cohort was 20.87% (95% CI: 15.8–25.9).

### 3.2 HCC patients

#### 3.2.1 HCC diagnosis coinciding with SARS‐CoV‐2 infection (de novo)

Fifty‐five patients had their first HCC diagnosis coincidentally with SARS‐CoV‐2 infection (54 HCC and one HCC‐iCC), 44 patients (80%) were cirrhotic. Their BCLC stage at SARS‐CoV‐2 infection was BCLC‐0 in 1 (1.8%), A in 22 (40.0%), B in 8 (14.5%), C in 14 (25.5%) and D in 10 (18.2%). In the BCLC‐A stage, there were 19 (86.4%) patients with a single nodule and 3 (13.6%) patients with up to 3 nodules and up to 3 cm each.

### 3.3 HCC diagnosis prior to SARS‐CoV‐2 infection

One hundred and sixty‐three (74.8%) patients had HCC history prior to SARS‐CoV‐2 infection. Their BCLC stage at SARS‐CoV‐2 infection was BCLC‐0 in 11 (6.8%), A in 48 (29.5%), B in 43 (26.4%), C in 44 (27.0%) and D in 17 (10.4%). In the BCLC‐A stage, there were 32 (66.7%) patients with a single nodule and 16 (33.3%) patients with up to 3 nodules and up to 3 cm each. Twenty (12.3%) patients had been treated with resection, 77 (47.2%) with loco‐regional treatments, 44 (27%) with systemic treatments, 17 (10.4%) were on Best Supportive Care (BSC) and 1 (0.6%) patient was being evaluated for liver transplantation.

Sixty‐nine (42.3%) of the 163 patients with prior HCC diagnosis and with established cancer treatment plan had to stop treatment or had it delayed because of SARS‐CoV‐2 infection. Forty‐four (63.8%) of these patients, restarted treatment after the resolution of the infection.

From the diagnosis of SARS‐CoV‐2 infection, the median follow‐up was 7.20 [2.20–10.79] months, 53 (33.7%) patients with a history of HCC developed HCC progression: new intra‐hepatic lesion in 21 (39.6%), growth of intra‐hepatic lesions in 16 (30.2%), new extra‐hepatic lesions in 12 (22.6%), and growth of extra‐hepatic lesions in 4 (7.6%) patients.

### 3.4 30‐day mortality rate in HCC patients

Forty (18.4%) patients died within the 30‐days of SARS‐CoV‐2 infection. Table 2 shows the 30‐day mortality rate according to the history of HCC, Child‐Pugh class and cause of death. The 30‐day mortality rate was 12.96% (95% CI 4.00–21.92) in *de‐novo* HCC patients and 20.25% (95% CI 14.08–26.41) in those with HCC history. It was 14.42 (95% CI 7.67–21.18), 16.11% (95% CI 6.96–25.25) and 52.94% (95% CI 29.21–76.67), in Child‐Pugh A, B and C patients respectively. Table S3 shows the 30‐day mortality rate according to the presence of cirrhosis.

**TABLE 2:** 30‐day mortality rate in HCC patients

	Events	Patients at risk	Mortality rate (95% CI)	*p*‐value
According to history of HCC				
de novo HTC	7	55	12.96 (4.00–21.92)	0.2237
History of HCC	33	163	20.25 (14.08–26.41)	
According to Child‐Pugh score^a^ ^,^ ^b^				
A	15	104	14.42 (7.67–21.18)	0.0005
B	10	63	16.11 (6.96–25.25)	
C	9	17	52.94 (29.21–76.67)	

	Events	Competing risks	Patients at risk^c^	Rate (95% CI)
According to cause of death^b^				
SARS‐CoV‐2 related	32	8	218	14.74 (10.39–19.8)
Non‐SARS‐CoV‐2 related	8	32	218	3.69 (1.73–6.83)

Abbreviations: 95% CI, 95% confidence interval; HCC, hepatocellular carcinoma; iCCA, intrahepatic cholangiocarcinoma; SARS‐CoV‐2, severe acute respiratory syndrome coronavirus 2.

^a^ Six non‐cirrhotic patients not included.

^b^ Includes de novo HCC and history of HCC patients.

^c^ One patient with HCC‐iCCA.

The 30‐day mortality was 14.74% (95% CI 10.39‐19.8) in the SARS‐CoV‐2‐related deaths using non‐SARS‐CoV‐2‐related deaths as competing risks, and 3.69% (95% CI 1.73–6.83) in the non‐SARS‐CoV‐2‐related deaths, using SARS‐CoV‐2‐related deaths as competing risks (Table 2).

The 30‐day mortality rate, considering non‐SARS‐CoV‐2‐related deaths as competing risks, increased along with the BCLC stage: 0/A 6.10% (95% CI 2.24–12.74), B 11.76% (95% CI 4.73–22.30), C 20.69% (95% CI 11.35–31.96) and D 34.52% (95% CI 17.03–52.78); *p* = .0017. The same effect persisted even after excluding the BCLC‐D patients (*p* = .0313). Table 3 shows the results of the competing risk Cox regression models that expose a sub‐distribution of the Hazard Ratio (HR) of 1.45 (95% CI 0.49–4.31; *p* = .5032) in BCLC‐B versus 0/A, and of HR = 3.13 (95% CI 1.29–7.62; *p* = .0118) in BCLC‐C versus 0/A.

**TABLE 3:** 30‐day SARS‐CoV‐2‐related death mortality rate according to BCLC stage

BCLC stage^a^	Events^b^	Competing events^c^	Patients at risk^e^	30‐day mortality rate, %(95% CI)	*p* ^d^	p‐value BCLC‐D excluded^d^	HR (95% CI)	*p*
0 or A	5	1	82	6.10 (2.24–12.74)	.0017	0.0313	Ref.	
B	6	1	51	11.76 (4.73–22.30)			1.45 (0.49–4.31)	.5032
C	12	0	58	20.69 (11.35–31.96)			3.13 (1.29–7.62)	.0118
D	9	6	27	34.52 (17.03–52.78)			‐	
Total	32	8	218					

Abbreviations: 95% CI: 95% confidence interval; BCLC: Barcelona Clinic Liver Cancer; HR: hazard ratio; SARS‐CoV‐2: severe acute respiratory syndrome coronavirus 2.

^a^ At SARS‐CoV‐2 diagnosis.

^b^ 30‐day SARS‐CoV‐2‐related deaths.

^c^ 30‐day non‐SARS‐CoV‐2‐related deaths.

^d^ Grey's test.

^e^ One patient HCC‐iCCA.

Eight patients had non‐SARS‐CoV‐2‐related deaths during the first 30‐day period. Table 4 describes the main causes of death. Six out of nine (75%) were BCLC‐D when infected and all but 1 died because of acute on chronic liver failure or HCC progression.

**TABLE 4:** 30‐day non‐SARS‐CoV‐2‐related causes of death in HCC patients

Cause of death	*n* (%)	BCLC stage (*n*)^a^
HCC progression	2 (25)	B (1), D (1)
Decompensated cirrhosis with HCC progression	2 (25)	D (2)
Decompensated cirrhosis without HCC progression	1 (12.5)	D (1)
Acute‐on‐Chronic liver failure	2 (25)	A (1), D (1)
Other^b^	1 (12.5)	D (1)
TOTAL	8 (100)	

Abbreviations: BCLC, Barcelona clinic liver cancer; HCC: hepatocellular carcinoma.

^a^ At the time of SARS‐CoV‐2 diagnosis.

^b^ Other: 1 patient died because of liver transplant rejection (BCLC‐D).

### 3.5 iCCA patients

Twenty‐six patients had prior diagnosis of iCCA and 6 were diagnosed coincidentally with SARS‐CoV‐2 infection.

The cancer stage according to the TNM 8th edition at the time of SARS‐CoV‐2 infection of patients with coincidentally iCCA diagnosis was IA in 1 (16.7%), IIIB in 1 (16.7%) and IV in 4 (66.6%) patients. On the other hand, cancer stage in patients with iCCA history was IA in 4 (15.4%), IB in 2 (7.7%), II in 2 (7.7%), IIIA in 1 (3.8%), IIIB in 7 (26.9%) and IV in 10 (38.5%) patients.

Of the 32 patients with iCCA diagnosis, 19 (59.4%) died; 12 (63.2%) were SARS‐CoV‐2‐related deaths and 7 (36.8%) were non‐SARS‐CoV‐2‐related.

### 3.6 iCCA diagnosis prior to SARS ‐CoV‐2 infection

Ten (38.5%) of the 26 patients with prior iCCA diagnosis and with an established cancer treatment plan had to stop or delayed it because of SARS‐CoV‐2 infection. Only 2 (20%) of these patients, restarted iCCA treatment after the resolution of the infection.

Table 1 describes the profile of these 26 patients.

During a median of 2.43 (0.33–8.78) months of follow‐up from the diagnosis of SARS‐CoV‐2 infection, 10 (38.5%) patients with a history of iCCA developed tumour progression.

## 4 DISCUSSION

To the best of our knowledge, this is the largest cohort of liver cancer patients infected with SARS‐CoV‐2 around the world. Our data are complementary to Iavarone et al.^10^ and Kim et al. publications.^15^ Both cohorts were focused on patients with liver disease history but only 11 and 19 HCC patients were included respectively. In addition, the present cohort is the first that describes the outcome of de‐*novo* liver cancer patients in whom the diagnosis was done during the SARS‐CoV‐2 infection. Lastly, despite there are no information in the literature about SARS‐CoV‐2 and cholangiocarcinoma and we are reporting the largest cohort of infected iCCA patients, the results should be considered only as descriptive because of the low number of patients included (*n* = 32). This could see as a limitation of the study but we would like to highlight the lack of data of iCCA in the literature and mention that is the largest cohort in this field.

Our study showed the 30‐day mortality rate of HCC patients who were under different cancer treatments during the first wave of the SARS‐CoV‐2. Nevertheless, as the SARS‐CoV‐2 infection could be acquired after being fully vaccinated,^16^, ^18^ these results could be used as reference for the evolution of HCC patients who are infected by SARS‐CoV‐2 because of non‐vaccination or waning immune defence.

As shown, the 30‐day mortality rate was increased along the BCLC stage (*p* = .0017) and that increment was maintained even when the BCLC‐D patients, who have a median survival lower than 3 months, were excluded (*p* = .0313). HCC progression or liver‐related deaths were the causes of non‐SARS‐CoV‐2‐related deaths in all of the 8 patients who died within the first month. Based on this information, it can be suggested that the non‐SARS‐CoV‐2‐related deaths were associated with the impact of the SARS‐CoV‐2 infection in the liver function or because of the result of stopping/delaying HCC treatment. It is already known that infections are events related to death in cirrhotic patients because of acute‐on‐chronic liver failure. However, Iavarone et al. reported that the 30‐day mortality rates were higher in patients with cirrhosis and COVID‐19 than in those with bacterial infections.^10^ Our results on the rate of 30 day‐mortality rate death according to the BCLC stage as well as the causes of non‐SARS‐CoV‐2‐related deaths reinforce the importance of characterising the effect of this new infection on the HCC patient's outcome.

For this reason, this study adds valuable information for physicians at clinical practice and for clinical researchers at the clinical trial level. The 30‐day mortality rate was 12.96% (95% CI 4.00–21.92) in de novo HCC patients and 20.25% (95% CI 14.08–26.41) in those with HCC history, but because of the small sample size and because of the confounder introduced by the HCC stage at the time of infection these results should be considered only as descriptive.

Our results could be useful for clinicians to inform patients and families about HCC prognosis in the context of the SARS‐CoV‐2 infection. In accordance with our results, 33.7% of patients with a history of HCC developed HCC progression during the follow‐up, while 40 (18.4%) patients with HCC (de novo or history) died within the first 30 days. However, only four deaths were for HCC progression, three were BCLC‐D when infected and only one death was because of HCC progression when patients at end‐stage (BCLC‐D) were excluded. Additionally, the risk of 30‐day SARS‐CoV‐2‐related death was similar between BCLC‐0/A and B stage [HR = 1.45 (95% CI 0.49–4.31; *p* = .5032)] but was significantly different between BCLC‐C versus 0/A stage (HR = 3.13 [95% CI 1.29–7.62; *p* = .0118]). These results could be explained by the higher rate of liver dysfunction in the BCLC‐C stage and by the treatment received at that stage.

The results of this project may help the researchers at the time of analysing the results of the ongoing Clinical Trials where the included patients may have been infected with SARS‐CoV‐2. Indeed, this data can be used as a reference for designing Clinical Trials. Nowadays, the SARS‐CoV‐2‐related‐cirrhosis complication and/or HCC progression‐related death in the context of SARS‐CoV‐2 infection will have to be considered as new causes of early treatment discontinuation. Accordingly, the expected number of patients who will stop or delay oncologic treatments for the reasons mentioned above as well as the number of patients who will die because of SARS‐CoV‐2 or cirrhosis complication/HCC progression in the context of SARS‐CoV‐2 infection should be taken into account when the sample size is calculated in future research projects. Indeed, underestimating these new factors may negatively impact the accuracy of clinical trial assumption about expected events and needed sample size.

As the SARS‐CoV‐2 infection is slowly weaning at different rates around the world, the results that we present will be of historical importance. It is important to register the impact of worldwide events, as we did for liver cancer. A noteworthy result is that 24.4% of the patients had a coincidental and incidental liver cancer diagnosis originated from tests for SARS‐CoV‐2 infection, which is a reminder of the importance of screening programmes. Finally, our data might help further studies to describe the impact of SARS‐CoV‐2 vaccination and the change in mortality associated with the new strains on liver cancer patients with SARS‐CoV‐2 infection.

The retrospective nature of the study is associated with variability in the local policy for hospitalization and management of the SARS‐CoV‐2 infection. In addition, despite of the fact all patients with de novo liver cancer had viable tumour, the study did not have central revision of the image's technique to confirm the viability of the cancer in the cohort of patients with a history of liver cancer at the time of infecting with SARS‐CoV‐2. However, we registered BCLC stage at the time of the SARS‐CoV‐2 diagnosis independently of the previous HCC treatment.

## 5 CONCLUSIONS

This is the largest cohort of liver cancer patients infected with SARS‐CoV‐2. It characterizes the risk of 30‐day SARS‐CoV‐2 death. The results can be used as reference for informing about HCC prognosis in the context of the SARS‐CoV‐2 infection.

## CONFLICT OF INTEREST

S. Muñoz‐Martínez. Speaker fees from Bayer and travel funding from Bayer and Eisai. He received grant support from Bristol Myers Squibb and Celgene. V. Sapena. Travel grants from Bayer. Consultancy fees from LEO‐Pharma. A. Forner. Lecture fees from Bayer, Gilead, Roche, Boston Scientific and MSD; consultancy fees from Bayer, AstraZeneca, Roche, Boston Scientific, SIRTEX, Exact Science and Guerbert. J. Bruix. Consultancy: AbbVie, ArQule, Astra, Basilea, Bayer, BMS, Daiichi Sankyo, GlaxoSmithKline, Gilead, Kowa, Lilly, Medimune, Novartis, Onxeo, Polaris, Quirem, Roche, Sanofi‐Aventis, Sirtex, Terumo/Grants: Bayer and Ipsen. M. Sanduzzi‐Zamparelli. received speaker fees and travel grants from Bayer and BTG, MSD. M. Bouattour. Consultant, Advisory Board and Travel support: Bayer Pharma, Ipsen, BMS, Eisai, Roche, AstraZeneca, Sirtex Medical. M. El‐Kassas. Consulting fees from Roche and EVA Pharma. Payment honoraria for lectures from Inspire, Spimaco and Roche. Payment for expert testimony from EVA, Janssen and ROCHE. Travel support by Roche, MSD and EVA Pharma. President of Egyptian Association for Research and Training in HepatoGastroenterology (EARTH). C. Guedes Leal. Lectures fees from IPSEN, AstraZeneca, Roche and Bayer. T. Mocan. Received travel grants from Bayer. J‐C.Nault. Received research grant from Ipsen and Bayer. R. Pinheiro‐Alves. Payment of honoraria for lectures from Bayer, Abbvie and Sirtex Medical. H. L. Reeves. Cancer Research UK (CR UK) HUNTER Accelerator Award (C9380/A26813). Grant support for Helen Reeves, paid to Institution. Consulting fees from SIRTEX, and Boston Scientific (payments to institution). Payment honoraria for lectures from Bayer and Eisai. L. da Fonseca. Lectures fees from BMS, Roche and Bayer. D. J. Pinato. Received lecture fees from ViiV Healthcare and Bayer Healthcare and travel expenses from BMS and Bayer Healthcare; consulting fees for Mina Therapeutics, EISAI, Roche and AstraZeneca; received research funding (to institution) from MSD and BMS. M. Varela. Reports receiving consulting fees from Astra‐Zeneca, Bayer, Eisai‐MSD, BMS, Roche; lectures fees from Bayer, Boston, Gilead, Eisai‐MSD, AbbVie; travel fees from Astra‐Zeneca, Bayer. M. R. Alvares‐da‐Silva. Has received Research grants, advisory board or speaker for AbbVie, Bayer, Biolab, Intercept, Ipsen, Gilead, MSD, Novartis, Genfit and Roche. L. Rimassa. Reports receiving consulting fees from Amgen, ArQule, AstraZeneca, Basilea, Bayer, BMS, Celgene, Eisai, Exelixis, Genenta, Hengrui, Incyte, Ipsen, IQVIA, Lilly, MSD, Nerviano Medical Sciences, Roche, Sanofi, Servier, Taiho Oncology, Zymeworks; lectures fees from AbbVie, Amgen, Bayer, Eisai, Gilead, Incyte, Ipsen, Lilly, Merck Serono, Roche, Sanofi; travel fees from AstraZeneca; and institutional research funding from Agios, ARMO BioSciences, AstraZeneca, BeiGene, Eisai, Exelixis, Fibrogen, Incyte, Ipsen, Lilly, MSD, Nerviano Medical Sciences, Roche, Zymeworks. M. Lozano. Lectures and educational presentations: Abbvie. Travel/accommodations, meeting expenses covered by Bayer, Gilead, Abbvie. J. M. González Santiago. JMGS received funding from the Fondo de Investigaciones Sanitarias, Instituto de Salud Carlos III, Spain (PI19/00819). F. Tacke. Has received research grants from Allergan, Gilead, Inventiva and BMS (to his institution). M. Sala. Travel/ accommodation/meeting expenses: Bayer. Eisai. Speaker fees: Bayer. M. Anders. Received speaker honoraria from Bayer, Roche, LKM‐Biotoscana, RAFFO. A. Lachenmayer. Consultancy CAScination, Advisory Board Johnson&Johnson and Histosonics. F. Piñero. Received speaker honoraria from Bayer, Roche, LKM‐Biotoscana, RAFFO. Research Grants from INC Argentinean National Institute of Cancer, Roche. G. Cabibbo. Consultancy fees from Bayer, Ipsen and Eisai. M. Peck‐Radosavljevic. Consultant for Bayer, Eisai, Guerbet, Intercept, Ipsen, Lilly, Roche; Speaker fees from Bayer, BMS, Eisai, Intercept, Ipsen, Lilly, MSD, Roche. Vergara. Travel grants from Bayer, Gilead, MSD and Abbvie. Lectures sponsored by Gilead, Abbvie, Intercept, Eisai and MSD. Consultancy: Eisai. C. Braconi. Consulting honoraria from Incyres. S. Pascual. Consulting fees from Eisai and Roche. Honoraria for lectures from Eisai, Chiari, Bayer, Educational events Gilead. Travel funding from Gilead. Participation in monitoring Board or Advisory Board: Eisai and Roche. C. Perelló. Received speaker honoraria and travel grants from Bayer, Gilead Sciences and Eisai. V. Mello. Fee lectures from Bayer and Roche. C. Rodríguez‐Lope. Travel grants from Bayer. Lecture fees and advisory board from Eisai. J. Acevedo. R. Villani. Research grant from Abbvie. C. Holland. Honoraria for lectures from IPSEN and travel support from Roche. C.F. Theodoro. Received speaker fees from Bayer. Z. Sparchez. Speaker honoraria from Bayer, AbbVie and Neola (Gilead). Travel grants from Neola (Gilead), AbbVie, Bayer. D. E. Viera‐Alves. Payment of honoraria for lectures from Novartis and Pfizer. R. Watson. Cancer Research UK (CR UK) HUNTER Accelerator Award (C9380/A26813) salary support paid to institution. A. D'Alessio. Travel support from Roche. M. Iavarone. Received speaker honoraria from Bayer, Gilead Sciences, BMS, Janssen, Ipsen, MSD, BTG‐Boston Scientific, AbbVie, Roche, EISAI and was consultant for BTG‐Boston Scientific, EISAI, Bayer and Guerbet. M. Reig. Consultancy: Bayer‐Shering Pharma, BMS, Roche, Ipsen, AstraZeneca, Lilly, BTG and UniversalDX. Paid conferences: Bayer‐Shering Pharma, BMS, Gilead, Lilly, Roche and UniversalDX. Research Grants (from the Institution): Bayer‐Shering Pharma, Ipsen.

The rest of the authors declare no conflict of interests.

## Supporting information


Appendix S1
