# Supplementary material for: Outcome of liver cancer patients with SARS‐CoV‐2 infection: An International, Multicentre, Cohort Study
Source: Liver Int. 2022 Jun 23;42(8):1891–901. doi: 10.1111/liv.15320 (PMC9347559; doi:10.1111/liv.15320)
Supplement: Supplementary file 1 — Appendix S1 [file LIV-42-1891-s001.docx]

**Outcome of liver cancer patients with SARS-CoV-2 infection. An International, Multicenter, Cohort Study.**

**List of Authors:**

Sergio Muñoz-Martínez, Victor Sapena, Alejandro Forner, Jordi Bruix, Marco Sanduzzi-Zamparelli,José Ríos, Mohamed Bouattour, Mohamed El Kassas, Cassia Regina Guedes Leal, Tudor Mocan, Jean-Charles Nault, Rogerio Camargo Pinheiro Alves, Helen L. Reeves, Leonardo da Fonseca, Ignacio García-Juárez, David J. Pinato, María Varela, Saleh A. Alqahtani, Mario Reis Alvares-da-Silva, Juan C. Bandi, Lorenza Rimassa, Mar Lozano, Jesús Manuel González Santiago, Frank Tacke, Margarita Sala, María Anders, Anja Lachenmayer, Federico Piñero, Alex França, Maria Guarino, Alessandra Elvevi, Giuseppe Cabibbo, Markus Peck-Radosavljevic, Ángela Rojas, Mercedes Vergara, Chiara Braconi, Sonia Pascual, Christie Perelló, Vivianne Mello, Carlos Rodríguez-Lope, Juan Acevedo, Rosanna Villani, Clemence Hollande, Valérie Vilgrain, Ahmed Tawheed, Carmem Ferguson Theodoro, Zeno Sparchez, Lorraine Blaise, Daniele Evaristo Viera-Alves, Robyn Watson, Flair José Carrilho, Carlos Moctezuma-Velázquez, Antonio D'Alessio, Massimo Iavarone and Maria Reig.

**Table of contents:**

| **Page** | **Title** |
| --- | --- |
| **2 – 4** | **Supplementary Table S1.** Centers included in the study. |
| **5** | **Supplementary Table S2**. 30-day mortality rate in HCC patients according to cirrhosis status. |

**Supplementary Table 1.** Centers included in the study.

| **Continent** | **Country** | **Hospital** | **Patients included** |
| --- | --- | --- | --- |
| **Africa** | **Egypt** | Helwan University | 16 |
| **America** | **Argentina** | Hospital Aleman | 3 |
|  |  | Hospital Universitario Austral | 3 |
|  |  | Hospital Italiano | 5 |
|  | **Brazil** | AMO CLINIC | 1 |
|  |  | Federal University of Sergipe | 2 |
|  |  | Hospital de Clínicas de Porto Alegre | 7 |
|  |  | Hospital das Clínicas. University of Sao Paulo School of Medicine | 12 |
|  |  | Hospital do Servidor Publico Estadual de São Paulo | 12 |
|  |  | Hospital Federal dos Servidores do Estado | 16 |
|  | **Mexico** | National Institute of Medical Sciences and Nutrition Salvador Zubirán | 11 |
| **Asia** | **Saudi Arabia** | King Faisal Specialist Hospital & Research Center | 8 |
| **Europe** | **Austria** | Klinikum Klagenfurt am Wörthersee | 2 |
|  | **France** | Jean Verdier Hospital | 15 |
|  |  | Beaujon University Hospital | 27 |
|  | **Germany** | Charité- University medicine Berlin | 4 |
|  | **Italy** | University of Foggia | 1 |
|  |  | San Gerardo Hospital | 2 |
|  |  | University of Naples Federico II | 2 |
|  |  | University of Palermo | 2 |
|  |  | IRCCS Humanitas Research Hospital | 5 |
|  |  | Foundation IRCCS Ca’ Granda Ospedale Maggiore Policlinico | 8 |
|  | **Romania** | Octavian Fodor Institute for Gastroenterology and Hepatology | 15 |
|  | **Spain** | HGU Alicante | 1 |
|  |  | Hospital Universitario Marqués de Valdecilla | 1 |
|  |  | University Hospital Puerta de Hierro | 1 |
|  |  | Instituto de Biomedicina de Sevilla- Hospital Virgen del Rocío | 2 |
|  |  | Parc Tauli Sabadell | 2 |
|  |  | Hospital Doctor Josep Trueta | 4 |
|  |  | Hospital Universitario Infanta Leonor | 5 |
|  |  | Hospital Universitario de Salamanca, IBSAL | 5 |
|  |  | Hospital Universitario Central de Asturias | 10 |
|  |  | Hospital Clinic de Barcelona | 12 |
|  | **Switzerland** | Inselspital, Bern University Hospital | 3 |
|  | **United Kingdom** | Beatson West of Scotland Cancer Centre / University of Glasgow | 1 |
|  |  | University Hospitals Plymouth NHS Trust | 1 |
|  |  | Imperial College London | 11 |
|  |  | Newcastle Hospitals NHS Foundation Trust | 12 |
| **TOTAL** | | | **250** |

**Supplementary Table 2.** Treatments used for SARS-CoV-2 indication.

| **SARS-CoV-2 treatment** | **n = 108** |
| --- | --- |
| Azithromycin | 53 (49.1) |
| Steroids | 46 (42.6) |
| Hydroxychloroquine | 42 (38.9) |
| Other Antibiotic | 36 (33.3) |
| Anticoagulation | 27 (25.0) |
| Oxygen | 21 (19.4) |
| Oseltamivir | 18 (16.7) |
| Lopinavir/ritonavir | 8 (7.4) |
| Tocilizumab | 5 (4.6) |
| Remdesivir | 4 (3.7) |
| ^†^Others | 6 (5.6) |

†Others: Ivermectin (2), Sarilumab vs Placebo in clinical trial (1),

unknown (1), hyperimmune plasma (1), Baricitinib (1)

**Supplementary Table 3.** 30-day mortality rate in HCC patients according to cirrhosis status.

| **Variable** | **% (95% CI)** |
| --- | --- |
| **Cirrhotic patients (n = 34)** | 18.27 (12.72 – 23.82) |
| **Non- cirrhotic patients (n = 6)** | 20.00 (5.69 – 34.31) |

HCC: Hepatocellular carcinoma; 95%CI: 95% Confidence interval
